# Epidermal Fatty Acid Binding Protein (E-FABP) Is Not Required for the Generation or Maintenance of Effector and Memory T Cells following Infection with *Listeria monocytogenes*

**DOI:** 10.1371/journal.pone.0162427

**Published:** 2016-09-02

**Authors:** Bing Li, Nathan W. Schmidt

**Affiliations:** Department of Microbiology and Immunology, University of Louisville, Louisville, KY, 40202, United States of America; University of Iowa, UNITED STATES

## Abstract

Following activation of naïve T cells there are dynamic changes in the metabolic pathways used by T cells to support both the energetic needs of the cell and the macromolecules required for growth and proliferation. Among other changes, lipid metabolism undergoes dynamic transitions between fatty acid oxidation and fatty acid synthesis as cells progress from naïve to effector and effector to memory T cells. The hydrophobic nature of lipids requires that they be bound to protein chaperones within a cell. Fatty acid binding proteins (FABPs) represent a large class of lipid chaperones, with epidermal FABP (E-FABP) expressed in T cells. The objective of this study was to determine the contribution of E-FABP in antigen-specific T cell responses. Following infection with *Listeria monocytogenes*, we observed similar clonal expansion, contraction and formation of memory CD8 T cells in WT and E-FABP-/- mice, which also exhibited similar phenotypic and functional characteristics. Analysis of *Listeria*-specific CD4 T cells also revealed no defect in the expansion, contraction, and formation of memory CD4 T cells in E-FABP-/- mice. These data demonstrate that E-FABP is dispensable for antigen-specific T cell responses following a bacterial infection.

## Introduction

CD4 and CD8 T cells are key components of the adaptive immune response through their ability to orchestrate the activity of multiple immune cell subsets and through the identification and elimination of infected cells. Antigen-specific CD4 and CD8 T cells for a defined epitope comprise a very small number amongst all the T cells in a host, ranging from several dozen to a couple thousand cells per mouse [1, 2]. Following infection these antigen-specific T cells undergo clonal expansion into effector T cells followed by contraction in number and progression to memory T cells, which provide enhanced immunity upon re-exposure to the microbe [3].

The metabolic pathways that support the biological activity of T cells as they progress from naïve to effector and effector to memory T cells has been the subject of exciting investigation in recent years. Naïve T cells use oxidative phosphorylation of glucose-derived pyruvate or fatty acid oxidation as their primary sources to yield the optimal amount of ATP per molecule of glucose or fatty acid, respectively [4]. Following T cell receptor activation and appropriate co-stimulatory signals there is a dynamic shift in the metabolic pathways utilized by T cells to support both the energetic demands of the cell and provide the necessary macromolecules to support cell growth and proliferation. Activated T cells use glycolysis to provide ATP while increased glutaminolysis is used to provide substrates for the TCA cycle [5]. In place of generating ATP, the TCA cycle in activated T cells is used as a source of macromolecules for synthesizing membrane lipids and nucleic acids [5]. Lipid metabolism also changes within activated T cells shifting from fatty acid oxidation in quiescent T cells to predominately fatty acid synthesis [6]. As T cells progress to memory there is a shift in metabolism back towards oxidative phosphorylation as a source of energy. Indeed, fatty acid oxidation is important for the development of memory CD8 T cell populations [7].

Metabolism of fatty acids requires they be transported into the cell and once inside the cell be bound to a protein chaperon to facilitate movement within the cell. Fatty acid binding proteins (FABP) are a class of lipid chaperones that bind to intracellular lipids with high affinity [8]. To date, nine family members have been identified in mice and humans, with some degree of tissue specificity [9]. Although epidermal FABP (E-FABP) has been shown to be required for differentiation of naïve CD4 T cells into Th17 cells [10], the role of E-FABP in T cells remains largely unknown. Since fatty acid metabolism plays an important role in naïve, effector, and memory T cells we hypothesized that E-FABP-/- mice may have impaired antigen-specific T cell responses following infection with the bacterium *Listeria monocytogenes*. Yet, *Listeria*-specific CD4 and CD8 T cells in E-FABP-/- mice showed no defect in expansion or memory formation as compared to WT mice. These results demonstrate that E-FABP is dispensable for antigen-specific T cell responses following infection with *L*. *monocytogenes*.

## Materials and Methods

### Mice and infection

Wild-type (WT) and E-FABP-/- [10, 11] mice were bred and maintained at the University of Louisville Research Resource Facilities. E-FABP deficiency had no apparent effect on immune cell differentiation *in vivo* [12]. Mice were fed NIH-31 Modified Open Formula Mouse/Rat Irradiated Diet (Envigo 7913; Envigo, Indianapolis, IN) or Laboratory Autoclavable Rodent Diet 5010 (LabDiet, St. Lous, MO) and provided autoclaved municipal tap water to drink. Male mice 5–13 weeks old were used for experiments. Attenuated (*actA*-deficient) *Listeria monocytogenes* expressing chicken ovalbumin (referred to as Lm-OVA) was provided by John Harty and described previously [13]. Mice were infected with 5x10^6^ Lm-OVA via tail vein injection.

### Ethics statement

All mice were housed according to the policies of the Institutional Animal Care and Use Committee of the University of Louisville and all studies were performed in accordance with the recommendations in the Guide for the Care and Use of Laboratory Animals of the National Institutes of Health. The experiments performed with mice in this study were approved by the Institutional Animal Care and Use Committee (IACUC protocol #14121), University of Louisville Animal Welfare Assurance Number (A3586-01). Mice were monitored daily by laboratory and/or animal facility personnel. The overall health of the mice was evaluated using the Body Conditioning (BC) score method. Mice that reached BC1 were euthanized. Mice were euthanized by first anesthetizing them with isoflurane followed by cervical dislocation.

### Detection and analysis of *Listeria*-specific T cells

Spleens were removed on the indicated days post Lm-OVA infection and disrupted into single-cell suspensions. Spleen cells were treated with Tris-ammonium chloride to lyse red blood cells. Total *Listeria*-specific T cells were determined by intracellular cytokine staining for IFN-γ after 5 hours of stimulation at 37°C with 200 nM OVA_257-264_ or 5 mM LLO_190-201_ in the presence of brefeldin A (Biolegend, San Diego, CA, USA). For MHC I tetramer staining, cells were incubated 45 min at room temperature with MHC class I tetramers loaded with OVA_257-264_ (generously provided by Dr. John Harty, University of Iowa, Iowa City, IA) followed by anti-CD8 and anti-Thy1.2 antibodies for 15 min at room temperature and then fixed with Fixation Buffer (Biolegend, San Diego, CA, USA). For phenotypic analysis, after incubation with peptides cells were surface stained with the indicated antibodies then fixed and permeabilized with Fixation Buffer. Intracellular staining for IFN-γ, IL-2, and TNF were done in the presence of 1X Permeabilization Wash Buffer (Biolegend, San Diego, CA, USA). Cell surface expression of CD62L was detected by incubating cells with 0.1 mM TAPI-2 (Peptides International, Louisville, KY, USA) for 30 min prior to and during stimulation with peptides.

### Antibodies

The following antibodies were purchased from BioLegend (San Diego, CA): CD8 (clone 53–6.7)-PerCP/Cy5.5 and -PE, CD4 (clone RM4-5)-PerCP/Cy5.5, CD27 (clone LG.3A10)-PE/Cy7, CD43 (clone 1B11)-APC, CD62L (clone MEL-14)-PE, CD90.2 (Thy1.2) (clone 53–2.1)-FITC, CD127 (clone A7R34)-Brilliant Violet 421, KLRG1 (clone 2F1/KLRG1)-APC, IFN-γ (clone XMG1.2)-FITC, TNF (clone MP6-XT22)-PE, IL-2 (clone JES6-5H4)-Brilliant Violet 421, Rat IgG2a (clone RTK2758)-PE, -APC, -Brilliant Violet 421, Rat IgG2b (clone RTK4530)-Brilliant Violet 421, Rat IgG1 (clone RTK2071)-PE, Syrian Hamster IgG (clone SHG-1)-APC, Armenian Hamster (clone HTK888)-PE/Cy7.

### Statistical analysis

Data were analyzed for statistical significance by the Mann-Whitney test using Graphpad Prism 6 (version 6.0h).

## Results

### Absence of E-FABP has no effect on *Listeria*-specific CD8 T cells

**Fig 1:**
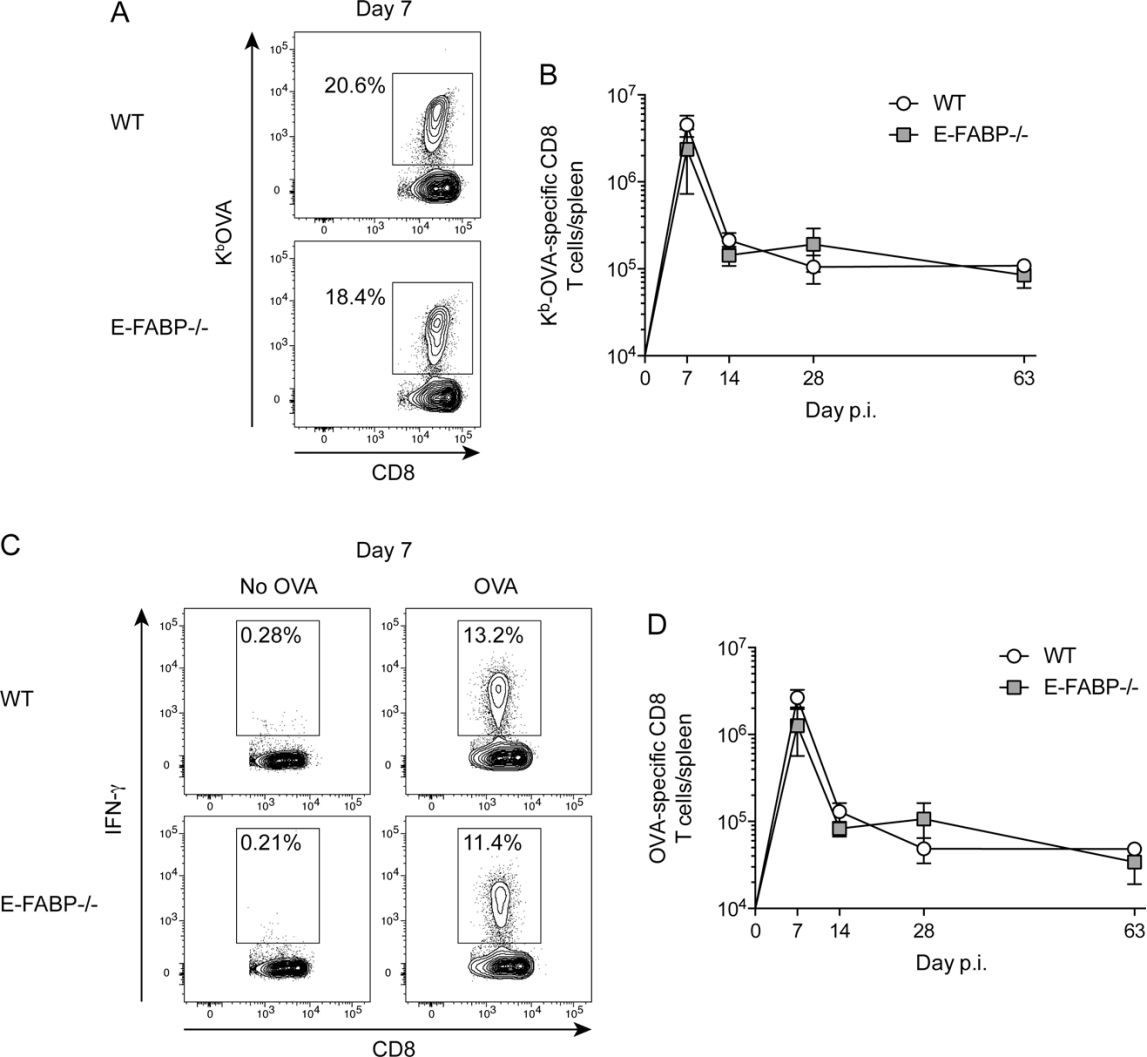
Kinetics of *Listeria*-specific CD8 T cells in WT and E-FABP-deficient mice. WT and E-FABP-/- mice were infected with 5x10^6^ Lm-OVA. OVA-specific CD8 T cells were detected by MHC class I tetramer (K^b^-OVA_257-264_) staining (A-B) or peptide (OVA_257-264_) stimulated intracellular cytokine staining for IFN-γ (C-D). (A and C) Representative contour plots showing the frequency of OVA-specific CD8 T cells, amongst all CD8+ cells, at the indicated days following infection with Lm-OVA. (B and D) Total number of OVA-specific CD8 T cells in the spleen. Data (mean±s.d.) are from 3-mice/time point/group except day 63 E-FABP-/- (n = 2). Data are representative of two independent experiments and were analyzed by the Mann-Whitney test.

To examine the role of E-FABP on antigen-specific T cell expansion, contraction, and development of memory T cells, WT and E-FABP-/- mice were infected with attenuated (*actA*-deficient) *Listeria monocytogenes* expressing chicken ovalbumin (Lm-OVA) [13]. Of note, based on transcript levels E-FABP is the predominant FABP expressed in naïve CD8 and CD4 T cells (data not shown). Following activation T cells switch metabolic activity from fatty acid oxidation to glycolysis [5]. One effect of this change in metabolism is increased capacity to make the effector cytokine IFN-γ [14]. It was unknown what effect deletion of E-FABP would have on T cell metabolism, and consequently effector cytokine production, following *Listeria* infection. Therefore, *Listeria*-specific CD8 T cells were quantified using both phenotypic detection via MHC I tetramers, which is independent of cytokine production, and functional detection via intracellular IFN-γ production following peptide stimulation. Following infection there were similar numbers of OVA-specific CD8 T cells, as detected by MHC I tetramers, at the peak of expansion (day 7), following contraction (day 14) and at both early (day 28) and late memory (day 63) time points in both WT and E-FABP-/- mice (Fig 1A and 1B). E-FABP-/- mice also had similar numbers of OVA-specific CD8 T cells compared to WT mice at all time points when detected by IFN-γ production (Fig 1C and 1D). Collectively, these data indicate that E-FABP is not required for production of IFN-γ in *Listeria*-specific CD8 T cells nor their expansion, contraction, and memory formation following infection.

**Fig 2:**
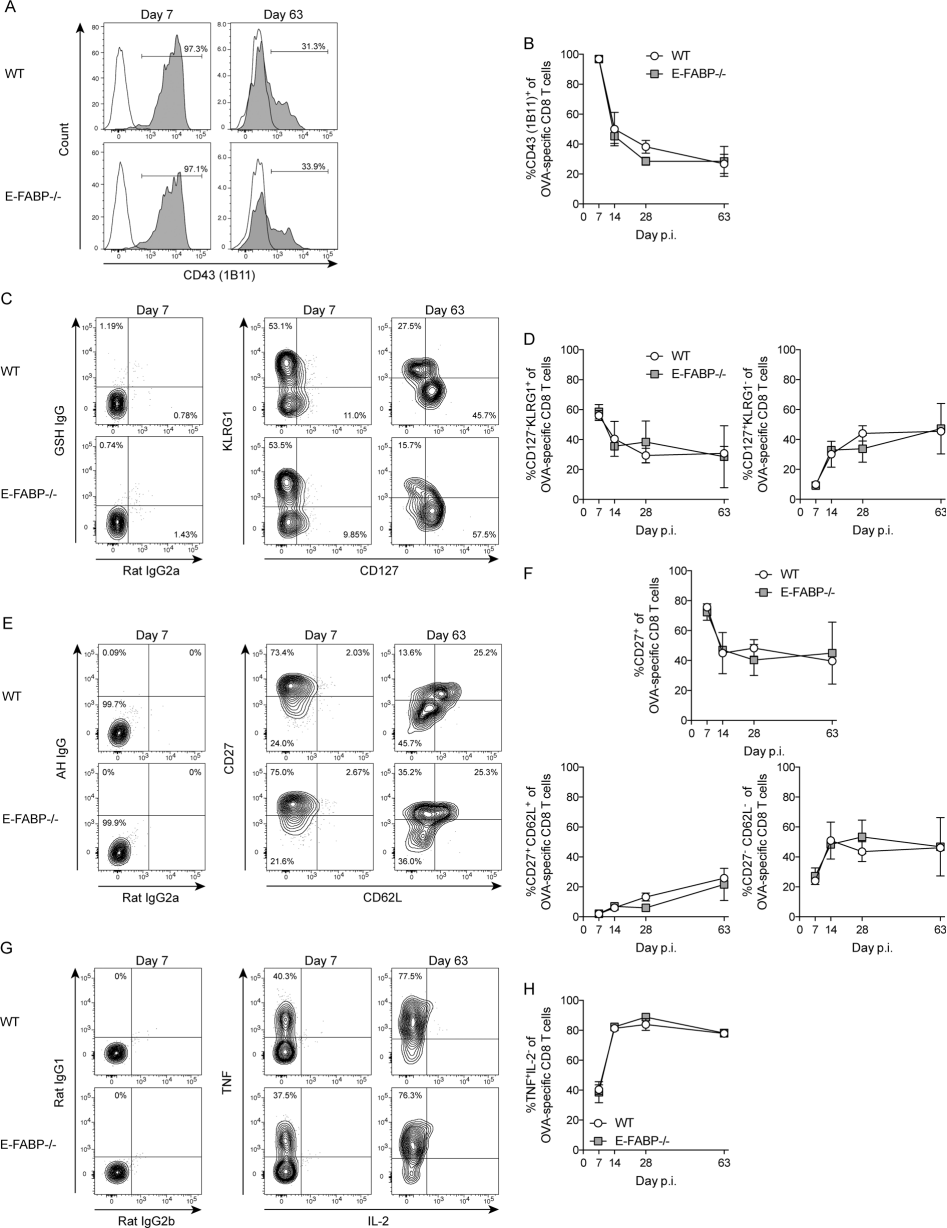
Phenotypic and functional characterization of *Listeria*-specific CD8 T cells. (A, C, E, G) Representative histograms and contour plots from day 7 or day 63 showing the frequency of OVA-specific CD8 T cells that are positive for the indicated cell surface marker or cytokine. Open histogram is isotype control; gray histogram is CD43 (clone 1B11). (B, D, F, H) Total percentage of OVA-specific CD8 T cells positive for the indicated cell surface marker or cytokine on the indicated days. Data (mean±s.d.) are from 3-mice/time point/group except day 63 WT (n = 2). Data are representative of two independent experiments and were analyzed by the Mann-Whitney test.

As antigen-specific CD8 T cells progress from effector to memory T cells they undergo defined phenotypic and functional changes. Effector CD8 T cells express high levels of core 2 O-glycan glycosylated CD43 that allow cells to enter inflamed tissues [15]. Analysis of core 2 O-glycan glycosylated CD43, via the 1B11 monoclonal antibody, revealed that OVA-specific effector (day 7) CD8 T cells in both WT and E-FAPB-/- mice expressed similar levels (Fig 2A and 2B). Antigen-specific T cells can also be distinguished based on differential expression of CD127 and KLRG1. CD127^-^KLRG1^+^ cells are defined as short-lived effector cells, while CD127^+^KLRG1^-^ cells are memory-precursor cells [16]. Consistent with similar numbers of both OVA-specific effector and memory CD8 T cells in WT and E-FABP-/- mice (Fig 1), there were similar fractions of CD127^-^KLRG1^+^ and CD127^+^KLRG1^-^ OVA-specific CD8 T cells at all time points analyzed (Fig 2C and 2D). Stable maintenance of memory CD8 T cell numbers involves homeostatic proliferation of central memory T cells that functionally progress to death-intermediate memory cells (T(DIM)), which are identified as CD62L^-^CD27^-^ [17]. Analysis of OVA-specific T(DIM) cells, in addition to all OVA-specific CD27^+^ cells and CD62L^+^CD27^+^ cells, revealed similar frequencies in all three of these populations in WT and E-FABP-/- mice (Fig 2E and 2F), which is consistent with maintenance of OVA-specific memory CD8 T cell numbers in both groups of mice (Fig 1). Finally, there were similar frequencies of TNF+ OVA-specific CD8 T cells in WT and E-FABP-/- mice at all time points while very little IL-2 production in both groups of mice (Fig 2G and 2H). Collectively, these data show that *Listeria*-specific CD8 T cells in WT and E-FABP-/- mice exhibit very similar phenotypic and functional characteristics.

### Absence of E-FABP has no effect on *Listeria*-specific CD4 T cells

**Fig 3:**
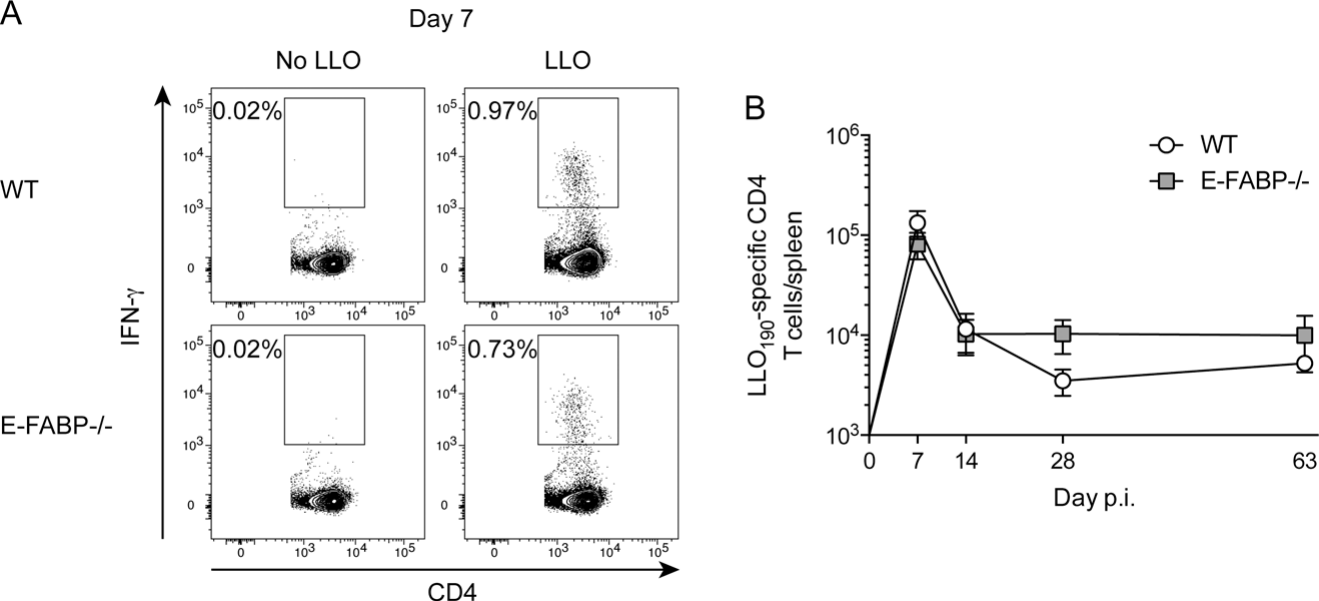
Kinetics of *Listeria*-specific CD4 T cells in WT and E-FABP-deficient mice. WT and E-FABP-/- mice were infected with 5x10^6^ Lm-OVA. (A) Representative contour plots showing the frequency of LLO-specific CD4 T cells as detected by peptide (LLO_190-201_) stimulated intracellular cytokine staining for IFN-γ at the indicated days following infection with Lm-OVA. (B) Total number of LLO-specific CD4 T cells in the spleen. Data (mean±s.d.) are from 3-mice/time point/group except day 63 WT (n = 2). Data are representative of two independent experiments and were analyzed by the Mann-Whitney test.

Prior studies have shown that different T cell subsets have varying requirements for fatty acid metabolism following activation [6, 18]. Consequently, antigen-specific CD4 T cells were also investigated following Lm-OVA infection in WT and E-FABP-/- mice (Fig 3). Following infection there were similar numbers of LLO-specific CD4 T cells on day 7 and 14 post infection in both groups of mice (Fig 3B). LLO-specific CD4 T cell numbers were also similar in WT and E-FABP-/- mice at both early (day 28) and late (day 63) memory time points (Fig 3B). These data demonstrate that E-FABP is also dispensable for antigen-specific CD4 T cells following infection with *L*. *monocytogenes*.

**Fig 4:**
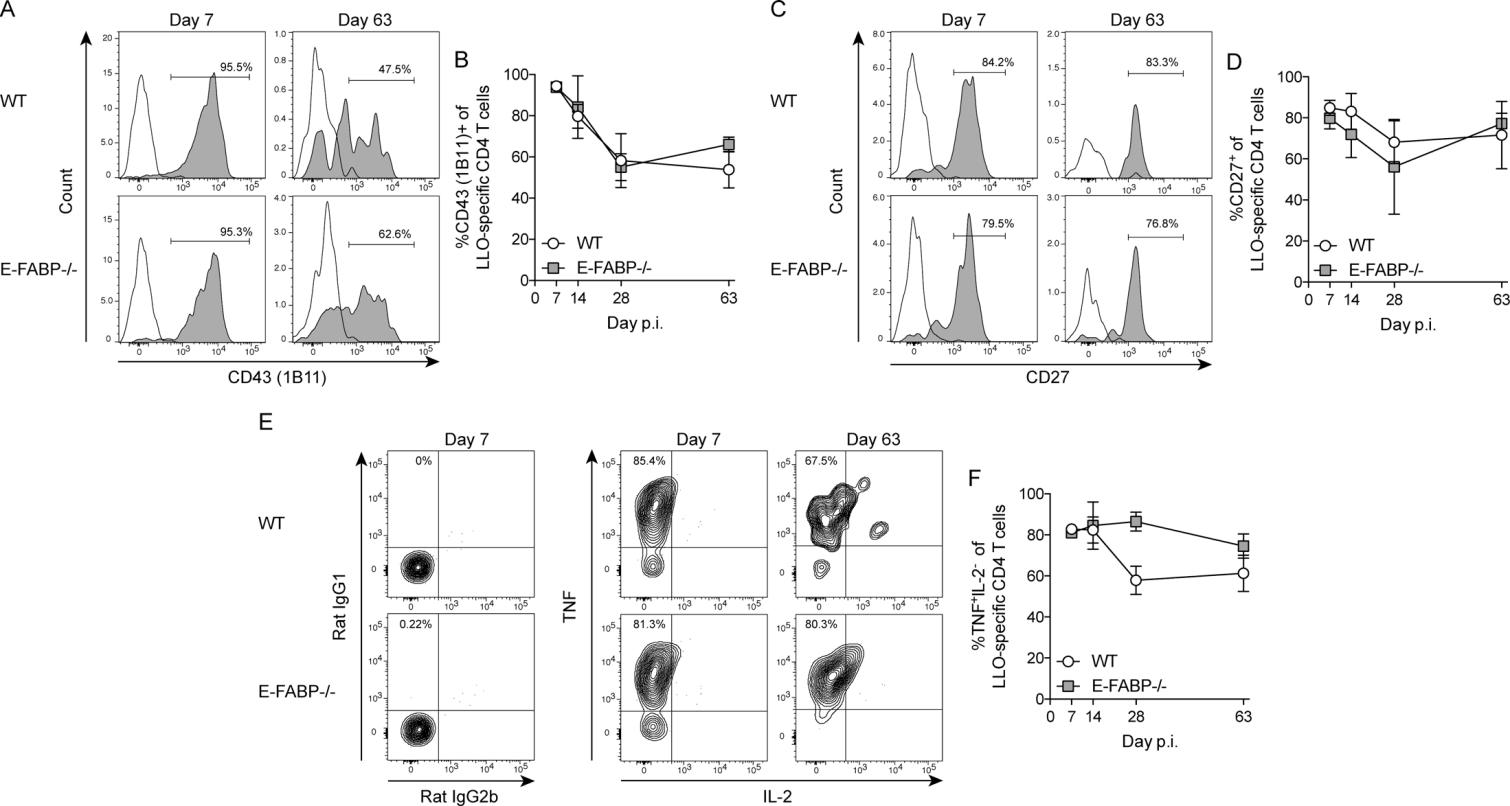
Phenotypic and functional characterization of *Listeria*-specific CD4 T cells. (A, C, E) Representative histograms and contour plots from day 7 or day 63 showing the frequency of LLO-specific CD4 T cells that are positive for the indicated cell surface marker or cytokine. (A) Open histogram is isotype control; gray histogram is CD43 (clone 1B11). (C) Open histogram is isotype control; gray histogram is CD27. (B, D, F) Total percentage of LLO-specific CD4 T cells positive for the indicated cell surface marker or cytokine on the indicated days. Data (mean±s.d.) are from 3-mice/time point/group except day 63 WT (n = 2). Data are representative of two independent experiments and were analyzed by the Mann-Whitney test.

The phenotypic and functional characterization of LLO-specific CD4 T cells was also evaluated in WT and E-FABP-/- mice following Lm-OVA infection. Consistent with OVA-specific CD8 T cells, LLO-specific effector CD4 T cells expressed high levels of glycosylated CD43 that declined through day 28 post infection with no difference in the expression level on LLO-specific CD4 T cells in either WT or E-FABP-/- mice (Fig 4A and 4B). Prior analysis has shown that CD27^+^ memory CD4 T cells exhibit elevated proliferative potential and survival compared to CD27^-^ memory CD4 T cells [19]. Consistent with the similar numbers of LLO-specific memory CD4 T cells in WT and E-FABP-/- mice (Fig 3), LLO-specific memory CD4 T cells in WT and E-FABP-/- mice had similar frequencies of CD27^+^ cells throughout the infection (Fig 4C and 4D). Finally, functional characterization of LLO-specific CD4 T cells following peptide stimulation revealed a similar capacity of cells to co-produce IFN-γ and TNF, while little IL-2, in WT and E-FABP-/- mice (Fig 4G and 4H). Collectively, these data demonstrate that following Lm-OVA infection; LLO-specific CD4 T cells in WT and E-FABP-/- mice have similar phenotypic and functional characteristics.

## Discussion

In this study, we demonstrated that E-FABP is not required for antigen-specific T cell responses following *L*. *monocytogenes* infection. Although E-FABP represents the predominant FABP family member expressed in naïve T cells, it is not known whether other FABP family members are dynamically expressed following T cell activation. These data emphasize the importance in examining the expression patterns of other FABP family members to assess functional redundancy in activated T cells.

Although our studies demonstrate that E-FABP is dispensable for effector and memory T cell responses, it has been previously shown to be important in the regulating the differentiation of CD4 T cells into IL-17 secreting Th17 cells [10]. In the absence of E-FABP there are elevated levels of the nuclear receptor peroxisome proliferator-activating receptor (PPAR)γ. Elevated levels of PPARγ blocked IL-6-induced STAT3 activation and downstream IL-21 production that subsequently stimulates the production of IL-17. Since there are no published reports attributing a role for PPARγ in *Listeria*-specific T cells, these data suggest the differential requirement of E-FABP in *Listeria*-induced T cell responses versus Th17 differentiation is likely attributed to E-FABP-dependent regulation of PPARγ activity.

Lipid metabolism is dynamically regulated following T cell activation shifting from primarily fatty acid oxidation to fatty acid synthesis. Sterol regulatory element-binding proteins (SREBPs), which control the lipid-biosynthesis program following T cell activation, are in part responsible for controlling the transition in lipid metabolism in activated T cells [20]. In the absence of SREBPs, CD8 T cells are impaired in growth and proliferation resulting in reduced clonal expansion of antigen-specific CD8 T cells following viral infection. The requirement for de novo fatty acid synthesis in effector CD8 T cell expansion was further demonstrated in mice deficient in acetyl-CoA carboxylase 1 (ACC1), which is involved in fatty acid synthesis [21]. ACC1-deficient CD8 T cells exhibit normal differentiation into effector CD8 T cells following bacterial infection; however they have reduced survival following activation resulting in impaired clonal expansion [18]. ACC1 is also important in activated CD4 T cells. ACC1-deficient CD4 T cells exhibit impaired Th1 and Th2 differentiation, and blocking both ACC1 and ACC2 with a pharmacological inhibitor reduced proliferation of CD4 T cells stimulated under Th1 and Th2 conditions [6]. Following either bacterial or viral infections, ACC2 was shown to be dispensable for both antigen-specific effector and memory CD8 T cells [22]. Collectively, these results demonstrate an important role for ACC1 in both CD8 and CD4 T cell responses, while ACC2 is dispensable in CD8 T cells. In contrast to effector T cells that undergo fatty acid synthesis, memory CD8 T cells require fatty acid oxidation for their generation and maintenance [7]. Surprisingly, memory CD8 T cells use de novo fatty acid synthesis to generate the lipids that are then used in fatty acid oxidation to generate energy [23]. This process is in part supported by the cytokine IL-7 [24]. IL-7 induces the expression of aquaporin 9, which is a glycerol transport [25, 26]. Aquaporin 9-deficient memory CD8 T cells had decreased import of glycerol and subsequently reduced synthesis of triacylglycerides, resulting in decreased survival of memory CD8 T cells [24].

Given the dynamic changes in lipid metabolism in effector and memory T cells it is likely lipid chaperones are important to this biology. Although these studies suggest that E-FABP is not required for lipid metabolism in CD4 and CD8 T cell homeostasis following bacterial infection, it remains unknown whether and how other lipid chaperones may contribute to regulating fatty acid metabolism. Given the important role of fatty acid metabolism in T cells it remains possible other FABP and/or other lipid chaperons may be important in regulating metabolic requirements of quiescent and activated T cells. Therefore, further studies into the characterization of these lipid chaperones will be important.
